# TNF-α/STAT3 pathway epigenetically upregulates Nav1.6 expression in DRG and contributes to neuropathic pain induced by L5-VRT

**DOI:** 10.1186/s12974-019-1421-8

**Published:** 2019-02-08

**Authors:** Huan-Huan Ding, Su-Bo Zhang, You-You Lv, Chao Ma, Meng Liu, Kui-Bo Zhang, Xiang-Cai Ruan, Jia-You Wei, Wen-Jun Xin, Shao-Ling Wu

**Affiliations:** 1 Neuroscience Program, Zhongshan School of Medicine, the Fifth Affiliated Hospital of Sun Yat-sen University, Sun Yat-sen University, Guangzhou, 510080 China; 2 Guangdong Provincial Key Laboratory of Malignant Tumor Epigenetics and Gene Regulation, Department of Rehabilitation Medicine, Sun Yat-sen Memorial Hospital, Sun Yat-sen University, 107 Yan Jiang West Road, Guangzhou, 510120 Guangdong China; 3 Department of Anesthesia and Pain Medicine, Guangzhou First People’s Hospital, Guangzhou Medical University, Guangzhou, 510120 China

**Keywords:** L5-VRT, TNF-α, STAT3, Nav1.6, Mechanical allodynia

## Abstract

**Background:**

Studies showed that upregulation of Nav1.6 increased the neuronal excitability and participated in neuropathic pain in the dorsal root ganglion (DRG). However, the molecular mechanisms underlying Nav1.6 upregulation were not reported yet.

**Methods:**

The paw withdrawal threshold was measured in the rodents following lumbar 5 ventral root transection (L5-VRT). Then qPCR, western blotting, immunoprecipitation, immunohistochemistry, and chromatin immunoprecipitation assays were performed to explore the molecular mechanisms in vivo and in vitro.

**Results:**

We found that the levels of Nav1.6 and phosphorylated STAT3 were significantly increased in DRG neurons following L5-VRT, and TNF-α incubation also upregulated the Nav1.6 expression in cultured DRG neurons. Furthermore, immunoprecipitation and chromatin immunoprecipitation assays demonstrated that L5-VRT increased the binding of STAT3 to the *Scn8a* (encoding Nav1.6) promoter and the interaction between STAT3 and p300, which contributed to the enhanced transcription of *Scn8a* by increasing histone H4 acetylation in *Scn8a* promoter in DRG. Importantly, intraperitoneal injection of the TNF-α inhibitor thalidomide reduced the phosphorylation of STAT3 and decreased the recruitment of STAT3 and histone H4 hyperacetylation in the *Scn8a* promoter, thus subsequently attenuating Nav1.6 upregulation in DRG neurons and mechanical allodynia induced by L5-VRT.

**Conclusion:**

These results suggested a new mechanism for Nav1.6 upregulation involving TNF-α/STAT3 pathway activation and subsequent STAT3-mediated histone H4 hyperacetylation in the *Scn8a* promoter region in DRG, which contributed to L5-VRT-induced neuropathic pain.

## Background

Chronic pain is a major health issue worldwide, especially in the patients with nerve injury [1, 2]. However, the effective treatment for neuropathic pain induced by nerve injury remains unsatisfactory [3], which is largely due to the lack of adequate understanding on the mechanism underlying neuropathic pain.

Voltage-gated sodium channel (VGSC) is a kind of multi-subunit transmembrane glycoprotein, which consists of nine members, named from Nav1.1 to Nav1.9 [4]. It is well known that these VGSCs play the critical roles in regulating the excitability and firing pattern of neurons and contribute to the neuropathic pain. For example, isoforms Nav1.7, Nav1.8, and Nav1.9 have been linked with pain disorders by regulating the action potential and firing properties of dorsal root ganglion (DRG) neurons [5, 6]. Moreover, our recent study shows that upregulation of Nav1.6 increased the DRG neuron excitability and contributed to the development of neuropathic pain [7]. However, the mechanism underlying the increased expression of Nav1.6 is not reported in neuropathic pain induced by nerve injury.

Evidence show that the inflammatory cytokines play an important role in the establishment and maintenance of chronic pain [8]. TNF-α is one of the cytokines with highlighted involvement in the pathogenesis of neuropathic pain. For example, nerve injury or chemotherapeutic drug treatment significantly increased the expression of cytokines TNF-α in the dorsal horn, and inhibition of TNF-α attenuated the chronic pain induced by nerve injury or the chemotherapeutic drug [9, 10]. In addition, many studies have revealed the potential interaction between ion channel and inflammatory cytokine, and their functional significance in the pathogenesis of neuropathic pain. For example, interleukin-6 increased the expression of acid-sensing ion channel 1a in rat articular chondrocytes [11] and application of cytokine TNF-α increased the sodium channel current in DRG neurons [12]. Furthermore, TNF-α could directly or indirectly regulate gene expression via JAK/STAT3 signaling pathway, and the activation of the STAT3 pathway-induced mechanical allodynia in rodents [13–15]. Our previous studies have demonstrated that TNF-α increases Nav1.6 expression in DRG [7]. However, it remains unknown whether activation of the TNF-α/STAT3 pathway mediates the Nav1.6 upregulation in DRG following lumbar 5 ventral root transection (L5-VRT).

## Methods

### Animals

Male Sprague-Dawley rats (200–220 g) and male wild-type (WT) C57 mice (20–30 g) were purchased from the Institute of Experimental Animals of Sun Yat-sen University. STAT3^flox/flox^ mice (ID: 016923) were obtained from the Jackson Laboratory. Animals were housed in separated cages and provided with food and water ad libitum, and the room was kept at 24 °C temperature and 50 to 60% humidity under a 12/12-h light/dark cycle. All experimental protocols were approved by the Local Animal Care Committee and were performed in accordance with the guidelines of the National Institutes of Health on animal care and the ethical guidelines. All efforts were made to minimize the number of animals used and their suffering.

### L5 ventral root transection (L5-VRT)

The L5-VRT model was performed as described previously [16]. Briefly, surgery and experimental procedures were performed on rats and mice after intraperitoneal administration of sodium pentobarbital (50 mg/kg, Sigma-Aldrich), and an L5 hemi laminectomy was performed to expose the L5 nerve root. The ventral root was gently pulled out with fine forceps and transected 2 to 3 mm proximal to the DRG, and a small portion (2 mm) of the root was dissected. In the sham group, an identical operation was performed for exposure of L5 ventral root, but the nerve was not transected.

### Drug administration and behavioral test

Recombinant rat TNF-α was obtained from R&D Systems (Minneapolis, MN, USA). Intraperitoneal injection of TNF-α synthesis inhibitor thalidomide (50 mg/kg; Sigma, USA) or intrathecal injection of STAT3 inhibitor S3I-201 (100 mg, 10 μL; Selleck Chemicals, USA) and TNF-α neutralizing antibody (10 μg/10 μL; R&D systems, Minneapolis, MN, USA) were performed 30 min before L5-VRT and for additional 9 days (total consecutive 10 days). Intrathecal injection was in accordance with the previously described method [17]. After the injection of sodium pentobarbital (50 mg/kg, i.p.), a polyethylene-10 catheter was inserted into the L5/L6 intervertebral subarachnoid space of the rat and the localization of the tip of the catheter was placed at the L5 spinal segmental level. The rats which exhibited hind limb paralysis or paresis after surgery were excluded from the study.

Von Frey hairs were used to assess the 50% withdrawal threshold as described previously [18]. In brief, each animal was loosely restrained in a plastic box on a metal mesh for three consecutive days (15 min/day) before testing. Von Frey filaments with different bending force were presented to the midplantar surface of each hind paw. In the absence of a paw withdrawal response to the initially selected hair, a stronger stimulus was presented, while in the event of paw withdrawal the next weaker stimulus was chosen. Optimal threshold calculation by this method required six responses in the immediate vicinity of the 50% threshold. The investigators who performed the behavioral tests were blinded to all treatments.

### Injection of adenovirus-associated vector

Recombinant adeno-associated virus encoding Cre and green fluorescent protein (GFP) marker (AAV8-Cre-GFP) and AAV encoding GFP (AAV8-GFP) were purchased from Beijing Vector Gene Technology Company Ltd. Four microliters (4 μL) of AAV8-Cre-GFP or AAV8-GFP was intrathecally injected in STAT3^flox/flox^ mice. pAAV-CAG-eGFP-U6-shRNA (Nav1.6) was commercially obtained from OBiO (Shanghai, China). The sequences of these shRNAs were presented as follows:

- shRNA1 5′-GGAAGACGCCATTGAAGAAGA-3′
- shRNA2 5′-GGAAGAATGTCAAGATCAACT-3′
- shRNA3 5′-GCAGATGGAGAACATTCTTTA-3′

These reagents were intrathecally delivered according to methods we previously described [17]. The experiments were performed after 21 days of the virus injection.

### Western blot

The L4 to L6 DRGs were immediately removed at different time points after application of sodium pentobarbital at 50 mg/kg dose (i.p.) and were homogenized on ice in 15 mM tris buffer containing the inhibitors of proteinase (Roche) and phosphatase (Roche) on ice. Protein samples were separated by SDS-PAGE and then transferred onto a PVDF membrane. The PVDF membrane was incubated with primary antibodies against Nav1.6 (1:200; Alomone labs), TNF-α (1:500; Santa Cruz), phosphorylated STAT3 (1:1000; Abcam), STAT3 (1:1000; Abcam), acetylated histone H3 (K9) (1:1000; Abcam), acetylated histone H4 (Ac-H4, 1:1000; Millipore), p300 (1:1000; Abcam), or β-actin (1:1500; CST), after blocking with the block buffer for 1 h at room temperature (RT). Enhanced chemiluminescence (ECL) (Pierce) was used to detect the immunocomplex after the blots were incubated with the secondary antibodies. The intensity of the band was examined by a computer-assisted imaging analysis system (ImageJ, NIH).

### Immunohistochemistry

Perfusion was immediately performed through the ascending aorta with 4% paraformaldehyde after being anesthetized by intraperitoneal injection of sodium pentobarbital (50 mg/kg). The DRG segments were removed and placed into the 4% paraformaldehyde for post-fixing overnight. Cryostat sections (16 μm) were cut, and immunohistochemistry was performed with primary antibody for Nav1.6 (1:100; Alomone labs), TNF-α (1:100, Santa Cruz), STAT3 (1:100; Abcam), phosphorylated STAT3 (Tyr705) (1:100; Abcam), IB4 (1:50; Sigma), GFAP (1:400; Chemicon), and NF-200 (1:200; Sigma). After incubation overnight at 4 °C, the sections were incubated with secondary antibodies, which conjugated with cy3 or FITC for 1 h at RT. To test the specificity of the TNF-α antibody, preincubation with TNF-α peptide was used [19]. Briefly, the TNF-α antibody was preincubated with TNF-α (R&D Systems, Inc.) in a concentration of 10 μg/mL, which was fivefold higher than that of anti-TNF-α antibody, in 4 °C for 1 h, and then the preincubated antibody was used for immunohistochemistry. The sections were examined with a Leica fluorescence microscope (Leica, Germany), and images were captured with a Leica DFC350 FX camera.

### DRG neurons culture

The ipsilateral DRG neurons of the procedure were dissociated by enzyme digestion as described previously with slight modifications [20]. In short, L4 to L6 DRGs were free from connective tissue sheaths and cut into small pieces with a pair of sclerotic scissors in Dulbecco’s modified Eagle’s medium (DMEM)/F12 medium (GIBCO, USA) under low temperature. The suspension of DRG neurons was then dissociated in DMEM/F12 medium supplemented with 10% fetal bovine serum after enzymatic and mechanical dissociation. Cells were cultured in a humidified atmosphere (5% CO_2_, 37 °C) and used for western blot within 24 h.

### RNA extraction and real-time quantitative polymerase chain reaction

A TRIzol reagent (Invitrogen) was used to extract total RNA from L4 to L6 DRGs. The reverse transcription was performed using Prime Script RT Master Mix (Takara). Real-time qPCR was processed with SYBR Premix Ex TaqII (Takara). The relative ratio of mRNA expression was quantified through the 2^−ΔΔCT^ method. The primer sequences of Nav1.6 were as follows: Forward 5′-TGGACGATACCAGCTCCTCA-3′, and Reverse 5′-ACGCAACCCTCTGTAAAGCA-3′.

### Chromatin immunoprecipitation (ChIP) assays

The ChIP assays were performed by a ChIP Assay Kit (Thermo). The L4 to L6 DRGs of rats were broken into pieces and placed in 1% formaldehyde for 2 min, then terminated with glycine. The DNA was subsequently digested by micrococcal nuclease after being broken via sonication. After the addition of ChIP dilution buffer, 100-μL samples were saved as input. The antibody of STAT3 (8 μL) or Ac-H4 (8 μL) was added to 500 μL for preclearing, and then the samples were incubated overnight at 4 °C. Immunoprecipitation with control rabbit IgG (Sigma) was performed as negative control. The DNA/protein/antibody complexes are purified into DNA by elution and reversion. QPCR was applied on 5 μL of precipitated DNA samples. Primers 5′-CTAGGCTGGGGACTTCTGTG-3′ and 5′-GCTAGTTTCCAGCAGGCAGT-3′ were projected to amplify a − 1530/− 1625 sequence, which localized on the *Scn8a* (encoding Nav1.6) promoter (which containing the STAT3 binding site) in rats. Finally, the relative ratio of CHIP/input was calculated.

### Coimmunoprecipitation (Co-IP)

Coimmunoprecipitation was performed using the Co-Immunoprecipitation Kit (Pierce). In brief, DRG tissues were excised quickly and homogenized in lysis buffer. The p300 antibody or STAT3 antibody, which was immobilized with resin, was used to collect the immune complexes. The eluted complexes from the resin were incubated and washed following the Kit manual and then analyzed by western blot using the STAT3 antibody or p300 antibody.

### Statistical analysis

Data were expressed as mean ± SEM and analyzed with SPSS 13.0. Western blot and qPCR were analyzed by independent Student’s *t* test and one-way analysis of variance (ANOVA). For the data of behavioral tests, one-way or two-way repeated-measures ANOVA were employed. The threshold for statistical significance was *P* < 0.05. Although no power analysis was performed, the sample size was determined according to previous publications in pain-associated behavior and molecular studies.

## Result

### Upregulation of Nav1.6 in DRG was involved in mechanical allodynia induced by L5-VRT

**Fig. 1:**
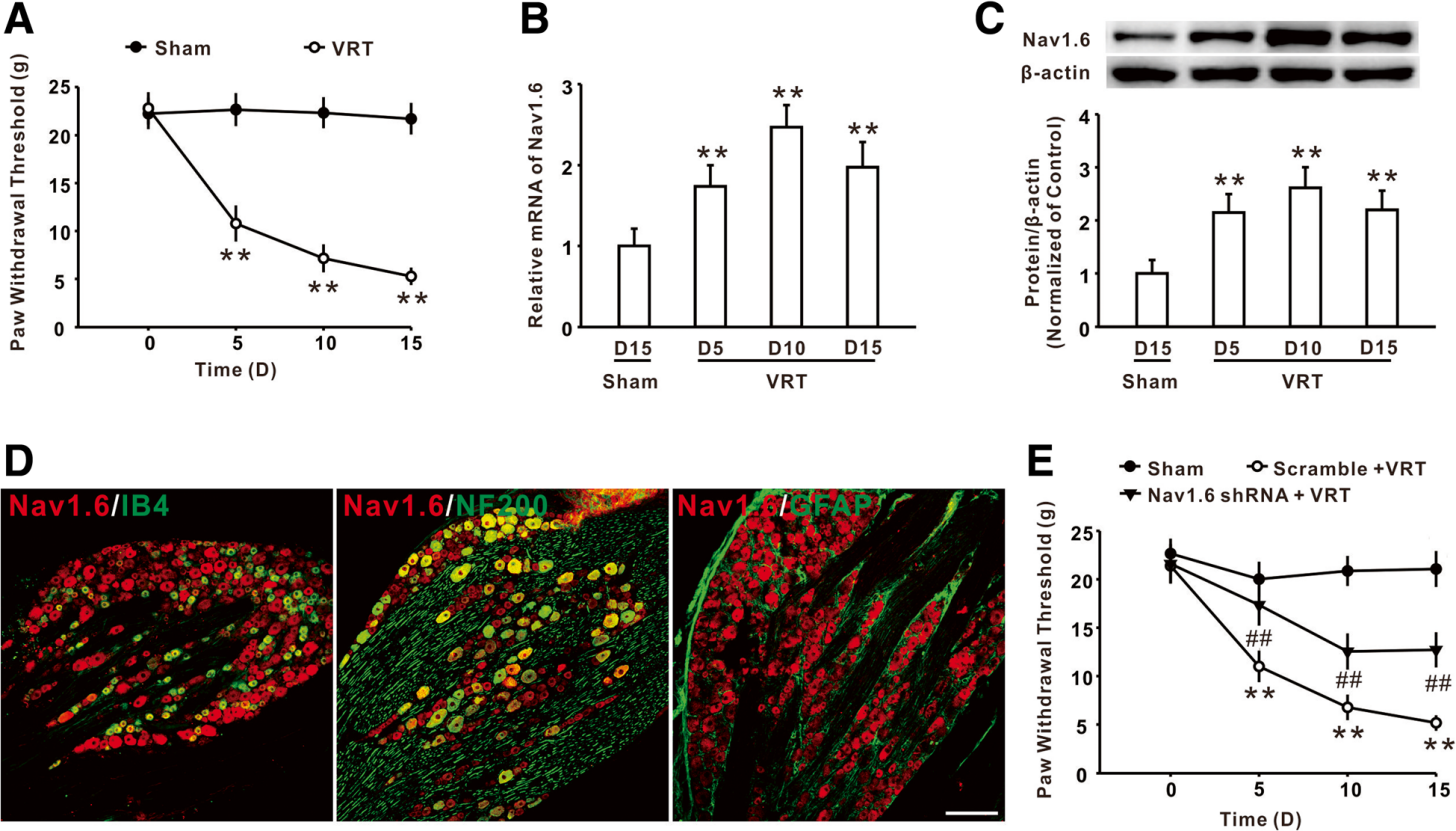
The increased expression of Nav1.6 in DRG was involved in L5-VRT-induced mechanical allodynia. **a** The hind paw withdraw threshold was substantially reduced by L5-VRT in rats. *n* = 12 per group, **P < 0.01 versus the sham group. **b** The expression of Nav1.6 mRNA was significantly increased following L5-VRT. *n* = 5 per group, **P < 0.01 versus the sham group. **c** Representative blots and histogram showed the effect of L5-VRT on Nav1.6 protein. *n* = 5 per group, **P < 0.01 versus the sham group. **d** The immunofluorescence staining showed the colocalization of Nav1.6 with IB4 and NF-200, but not GFAP, in DRG. *n* = 3 per group, Scale bar, 50 μm. **e** Application of Nav1.6 shRNA significantly attenuated L5-VRT-induced mechanical allodynia. *n* = 12 per group, **P < 0.01 versus the sham group, ##*P* < 0.01 versus the corresponding L5-VRT group

Consistent with our previous study [19, 21], the results showed that L5-VRT markedly decreased the withdrawal threshold compared to the sham group (Fig. 1a). The qPCR and western blot analysis demonstrated that nerve injury significantly increased the mRNA and protein level of Nav1.6 in DRG, which occurred on day 5 and persisted to day 15 (the experiment end) (Fig. 1b, c). Double immunostaining showed that Nav1.6 was primarily expressed in IB4- and NF-200-positive neurons, but not in GFAP-positive satellite glial cells (Fig. 1d). Importantly, intrathecal application of Nav1.6 shRNA, which have been shown to inhibit the expression of Nav1.6 protein in DRG in our previous study [7], attenuated the mechanical allodynia induced by L5-VRT (Fig. 1e). These results implied that the increased expression of Nav1.6 in DRG might be involved in the behavioral hypersensitivity induced by nerve injury.

### Increased TNF-α contributed to Nav1.6 upregulation and mechanical allodynia induced by L5-VRT

**Fig. 2:**
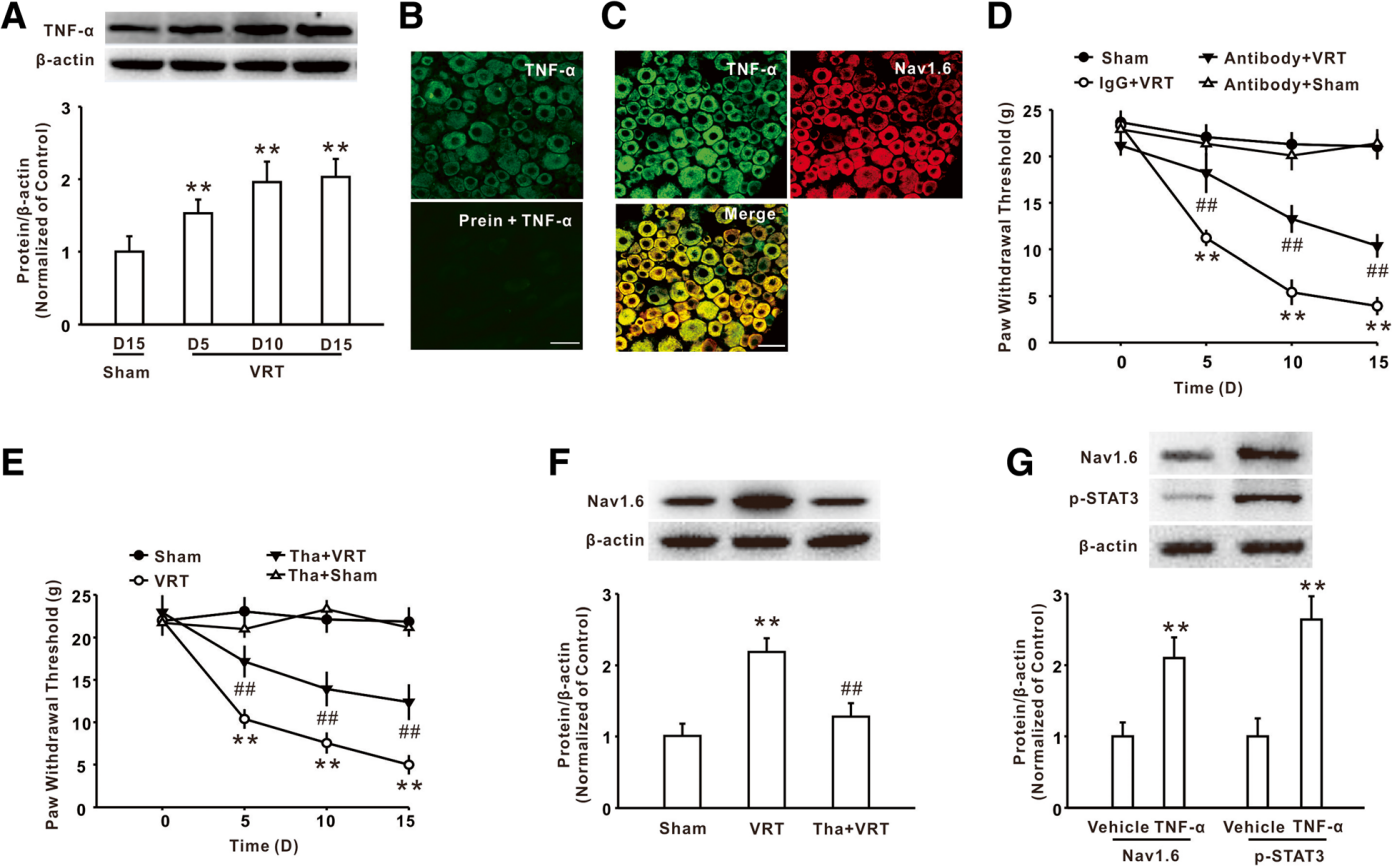
TNF-α mediated the increase of Nav1.6 expression following L5-VRT. **a** Representative blots and histogram showed the upregulation of TNF-α following L5-VRT. *n* = 5 per group, **P < 0.01 versus the sham group. **b** The fluorescence intensity by staining with anti-TNF-α antibody was lower in the sections than by preincubating the antibody with TNF-α peptide. *n* = 3 per group, Scale bar, 50 μm. **c** The double immunofluorescence staining showed the colocalization of TNF-α with Nav1.6. *n* = 3 per group, Scale bar, 50 μm. **d** Intrathecal injection of TNF-α neutralizing antibody significantly increased the withdrawal threshold in rats with L5-VRT. *n* = 12 per group, **P < 0.01 versus the sham group, ##*P* < 0.01 versus the corresponding L5-VRT group. **e** Continuous intraperitoneal application of the TNF-α inhibitor thalidomide significantly relieved L5-VRT-induced mechanical allodynia. *n* = 12 per group, **P < 0.01 versus the sham group, ##*P* < 0.01 versus the corresponding L5-VRT group. **f** Representative blots and histogram showed the expression of Nav1.6 was decreased after intraperitoneal application of the TNF-α inhibitor thalidomide. *n* = 5 per group, **P < 0.01 versus the sham group, ##*P* < 0.01 versus the corresponding L5-VRT group. **g** The incubation with TNF-α increased the expression of Nav1.6 and p-STAT3 in cultured DRG neurons. *n* = 6 per group, **P < 0.01 versus the vehicle group

Next, we found that L5-VRT also markedly induced the increase of TNF-α in DRG (Fig. 2a). Then, we performed the double immunostaining to determine the type of cell-expressing TNF-α. As shown in Fig. 2b, preincubation with TNF-α peptide significantly decreased the immunofluorescence in DRG section staining with an anti-TNF-α antibody, suggesting the specificity of the TNF-α antibody. The immunosignal of Nav1.6 was localized in TNF-α-positive cells (Fig. 2c). Notably, intrathecal injection of TNF-α neutralizing antibody significantly increased the withdrawal threshold in the rats with L5-VRT (Fig. 2d). Similarly, inhibition of TNF-α synthesis by intraperitoneal application of TNF-α inhibitor thalidomide at a dose of 50 mg/kg significantly inhibited mechanical allodynia (Fig. 2e) and Nav1.6 upregulation on day 15 was induced by L5-VRT (Fig. 2f). In vitro studies demonstrated that treatment with TNF-α enhanced the expression of Nav1.6 and p-STAT3 in cultured DRG neurons (Fig. 2g). Together, these results suggested that TNF-α played an extensive role in Nav1.6 upregulation in DRG neurons and mechanical allodynia following L5-VRT.

### TNF-α was involved in the activation of STAT3 following L5-VRT

**Fig. 3:**
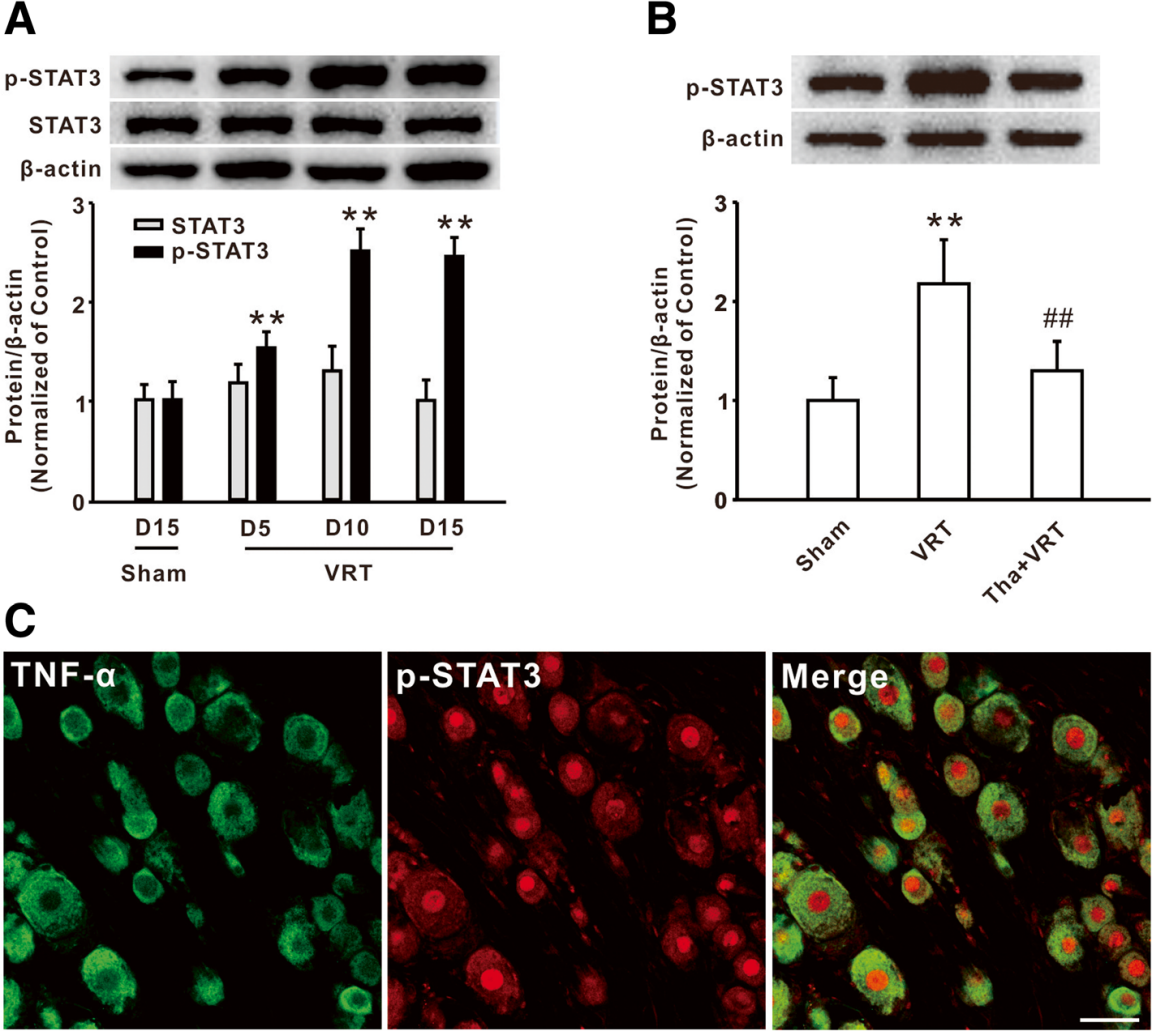
TNF-α mediated the upregulation of p-STAT3 following L5-VRT. **a** The upregulation of p-STAT3 was induced by L5-VRT. *n* = 5 per group, **P < 0.01 versus the sham group. **b** Representative blots and histogram showed the effect of thalidomide on the p-STAT3 upregulation in the rats with L5-VRT. *n* = 5 per group, **P < 0.01 versus the sham group, ##*P* < 0.01 versus the corresponding L5-VRT group. **c** The immunofluorescence staining showed the colocalization of TNF-α with p-STAT3. *n* = 3 per group, Scale bar, 50 μm

Our previous study demonstrated that activation of STAT3 in the spinal dorsal horn was involved in paclitaxel-induced neuropathic pain [17]. Here, we further investigated the function of STAT3 in DRG in the rodents with L5-VRT. Western blot analysis showed that the level of p-STAT3, but not total STAT3, was markedly increased in DRG on day 5 and persisted to day 15 following nerve injury (Fig. 3a). It is known that STAT3 is phosphorylated in response to cytokines such as IL-6 and IL-10 [22]. However, whether cytokine TNF-α is also involved in STAT3 activation in neuropathic pain remains unknown. Next, we observed the effect of the TNF-α inhibitor on the expression of p-STAT3 following L5-VRT. The western blot results showed that intraperitoneal injection of thalidomide (50 mg/kg) prevented the increase of p-STAT3 in DRG on day 15 following L5-VRT (Fig. 3b). The double immunostaining further showed that the expressions of p-STAT3 were localized in TNF-α-positive cells (Fig. 3c). Therefore, these results suggested that the increase of TNF-α contributed to the activation of STAT3 following L5-VRT.

### The STAT3 activation mediated by TNF-α participates in Nav1.6 upregulation in DRG

**Fig. 4:**
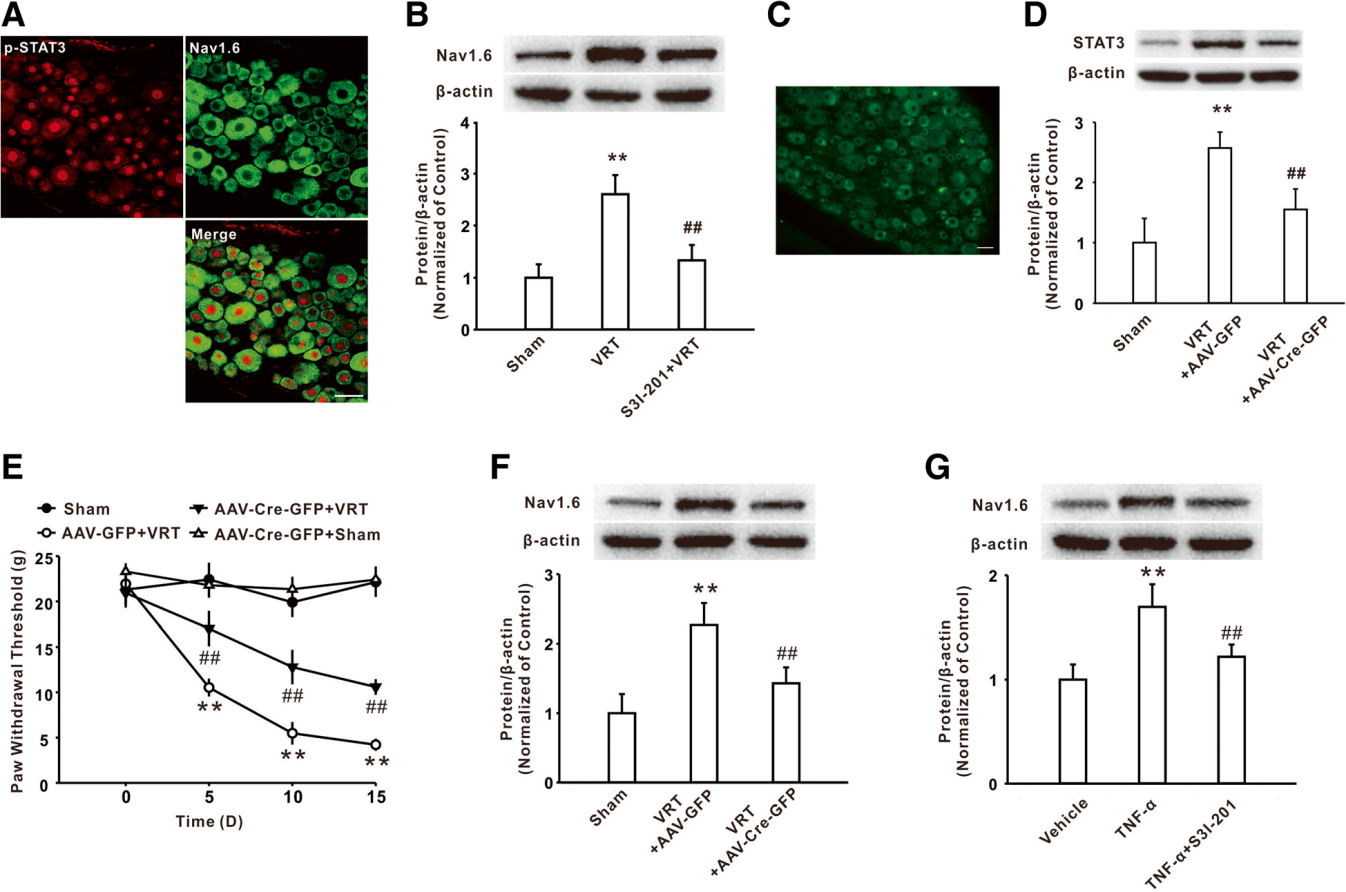
TNF-α via activating STAT3 upregulated Nav1.6 expression in DRG. **a** The immunofluorescence staining showed the colocalization of p-STAT3 with Nav1.6 in DRG. *n* = 3 per group, Scale bar, 50 μm. **b** The increase of Nav1.6 induced by L5-VRT was reduced by intrathecal injection of STAT3 activity inhibitor S3I-201. *n* = 5 per group, **P < 0.01 versus the sham group, ##*P* < 0.01 versus the corresponding L5-VRT group. **c** A marked green fluorescence in the restricted DRG of mice was observed on day 21 after AAV-Cre-GFP injection. *n* = 3 per group, Scale bar, 50 μm. **d** Intrathecal injection of AAV-Cre-GFP decreased the STAT3 expression in the STAT3^flox/flox^ mice. *n* = 5 per group, **P < 0.01 versus the sham group, ##*P* < 0.01 versus the corresponding L5-VRT group. **e** Mechanical allodynia induced by L5-VRT was inhibited in AAV-Cre-GFP-injected STAT3^flox/flox^ mice. *n* = 12 per group, **P < 0.01 versus the sham group, ##*P* < 0.01 versus the corresponding L5-VRT group. **f** Intrathecal injection of the AAV-Cre-GFP into the STAT3^flox/flox^ mice significantly decreased the Nav1.6 increase induced by L5-VRT. *n* = 5 rats per group, **P < 0.01 versus the sham group, ##*P* < 0.01 versus the corresponding L5-VRT group. **g** Increase of Nav1.6 induced by TNF-α incubation was reduced by S3I-201 in cultured DRG neurons. *n* = 6 per group, **P < 0.01 versus the vehicle group, ##*P* < 0.01 versus the corresponding TNF-α group

Next, we investigated whether STAT3 activation mediated the increases of Nav1.6 in DRG in rodent with L5-VRT. Firstly, the double immunostaining study showed that the immunoreactivity of Nav1.6 was expressed in p-STAT3-positive cells (Fig. 4a). Intrathecal injection of STAT3 activity inhibitor S3I-201 prevented the upregulation of Nav1.6 on day 15 following L5-VRT (Fig. 4b). Local deficiency of STAT3 in DRG by intrathecal injection of AAV-Cre-GFP into the STAT3^flox/flox^ mice (Fig. 4c, d) at 21 days prior to the procedure significantly attenuated the mechanical allodynia (Fig. 4e) and reduced the upregulation of Nav1.6 on day 15 following L5-VRT (Fig. 4f). Notably, pre-incubation of cultured DRG neurons with S3I-201 also significantly reduced the upregulation of Nav1.6 following incubation of TNF-α (Fig. 4g). All these results suggested that activation of STAT3 signaling was involved in TNF-α-induced Nav1.6 upregulation in DRG neurons.

### TNF-α promotes the combination of p-STAT3/p300 and *Scn8a* promoter in DRG

**Fig. 5:**
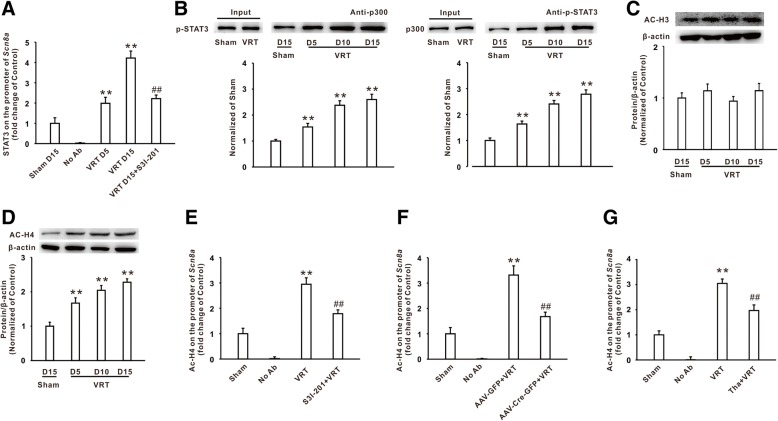
TNF-α via activating STAT3 enhanced the binding of p-STAT3 in the *Scn8a* promoter in DRG. **a** Chromatin immunoprecipitation assay showed the binding of p-STAT3 in the *Scn8a* promoter after L5-VRT. *n* = 6 per group, **P < 0.01 versus the sham group, ##*P* < 0.01 versus the corresponding L5-VRT group. **b** Increased p-STAT3 content was significantly immunoprecipitated by the p300 antibody, and increased p300 was also significantly immunoprecipitated by the p-STAT3 antibody on the different time points after L5-VRT. *n* = 5 per group, **P < 0.01 versus the sham group. **c** Representative blots and histogram showed the expression of acetylated histone H3 following L5-VRT. *n* = 5 per group. **d** Representative blots and histogram showed the expression of acetylated histone H4 after L5-VRT. *n* = 5 per group, **P < 0.01 versus the sham group. **e** Acetylation of H4 on the promoter region of *Scn8a* the flanking STAT3 binding site in chromatin immunoprecipitation assay in rats with S3I-201 treatment. *n* = 6 per group, **P < 0.01 versus the sham group, ##*P* < 0.01 versus the corresponding VRT group. **f** The change of histone H4 acetylation on the promoter region of *Scn8a* flanking the STAT3 binding site in the STAT3^flox/flox^ mice which were injected with AAV-Cre-GFP. *n* = 6 per group, **P < 0.01 versus the sham group, ##*P* < 0.01 versus the corresponding VRT group. **g** The effect of thalidomide (i.p.) on the histone H4 acetylation on the *Scn8a* promoter region following L5-VRT. *n* = 6 per group, **P < 0.01 versus the sham group, ##*P* < 0.01 versus the corresponding VRT group

As a member of the STAT family of transcription factors, STAT3 is now known to regulate the expression of many genes such as cytokine, anti-apoptotic, and pro-survival genes [23, 24]. Here, to determine whether the activated STAT3 signaling transcriptionally regulated the expression of Nav1.6, we first observed the binding of STAT3 in the *Scn8a* promoter in DRG using a ChIP-PCR assay. TFSEARCH and JASPAR database analysis showed that there was a potential potent binding site for STAT3 at the position of − 1534/− 1544 in the promoter region of *Scn8a*. The DNA, which was precipitated by the p-STAT3 antibody, was subjected to qPCR to amplify a 96-bp fragment (− 1530/− 1625) of the *Scn8a* promoter which contains the STAT3 binding site with the designed primers. The results of qPCR analysis revealed that the binding of STAT3 to the *Scn8a* promoter was enhanced in DRG on day 5 and day 15 following L5-VRT compared to the sham group, which can be reversed by the STAT3 activity inhibitor S3I-201 in the modeled rats (Fig. 5a). To further investigate the mechanisms underlying the regulation of Nav1.6 expression by STAT3, immunoprecipitation (IP) was performed in lysates from DRG tissue. The result showed that L5-VRT markedly increased p-STAT3 content on day 5, 10, and 15 in the immunocomplex precipitated by the p300 antibody (Fig. 5b). Similarly, the content of p300 precipitated by the p-STAT3 antibody was also augmented across different time points after L5-VRT (Fig. 5b). As it is well known, p300 plays an important role in the modulation of histone acetylation and chromatin remodeling, which contributes to the transcription of many inflammatory genes. Hence, we further examined whether L5-VRT changed the histone acetylation in the *Scn8a* promoter region in DRG. Firstly, western blot analysis showed that the total acetylation of histone H4 was increased significantly (Fig. 5d) while that of H3 did not obviously change after L5-VRT (Fig. 5c). Moreover, chromatin immunoprecipitation assay further found that L5-VRT induced the increase of acetylation of histone H4 in the *Scn8a* promoter region which contains the STAT3 binding site in DRG on day 15, and this H4 hyperacetylation can be reversed by S3I-201 in the modeled rats (Fig. 5e) or by injection with AAV-Cre-GFP in STAT3^flox/flox^ mice with L5-VRT (Fig. 5f). Importantly, the application of the TNF-α inhibitor thalidomide also prevented the increase of histone H4 acetylation in the *Scn8a* promoter region on day 15 induced by L5-VRT (Fig. 5g). Considering the TNF-α-mediated regulation of the p-STAT3 activity, these studies suggested an enhanced interaction between p-STAT3 and p300, possibly mediated by TNF-α, in the *Scn8a* promoter region, which increased the histone H4 acetylation and facilitated the Nav1.6 expression in the rodents with L5-VRT.

## Discussion

In the present study, we elucidated a novel molecular mechanism underlying the epigenetic upregulation of Nav1.6 by an enhanced TNF-α/STAT3 signaling pathway in DRG, which contributed to neuropathic pain induced by L5-VRT in the rodent models. The results showed that the expression of Nav1.6 and p-STAT3 was significantly increased in DRG neurons following L5-VRT. Incubation of TNF-α also upregulated the expression of Nav1.6 and p-STAT3 in cultured DRG neurons. Moreover, IP and ChIP assays showed that L5-VRT enhanced the interaction between p-STAT3 and p300 and increased the recruitment of p-STAT3 and histone H4 acetylation on the *Scn8a* promoter in DRG. Suppression of STAT3 activation with local knockdown or inhibitor S3I-201 significantly attenuated H4 acetylation, and Nav1.6 upregulation in DRG, and relieved mechanical allodynia induced by L5-VRT. Importantly, inhibition of the TNF-α activity by thalidomide inhibited the STAT3 activation, H4 acetylation, and Nav1.6 upregulation in DRG following L5-VRT. Taken together, these findings, for the first time, proved that the activation of the TNF-α/STAT3 pathway may be a novel mechanism for Nav1.6 upregulation in neuropathic pain induced by nerve injury.

Nav1.6, as an isoform of sodium channel, contributes to the neuron excitability and plays a critical role in the development of CNS diseases. For example, Nav1.6 upregulation is involved in neuropathic pain induced by SNL or diabetes in mice [25, 26]. The present study showed that Nav1.6 also played a critical role in L5-VRT-induced mechanical allodynia. Importantly, we further examined the mechanism underlying the Nav1.6 upregulation in neuropathic pain. STAT3, as a pivotal transcriptional factor, was involved in the process of chronic pain. For example, a marked activation of STAT3 in the spinal cord was observed after spinal nerve injury or chemotherapeutic drug treatment [13, 17], and inhibition of STAT3 attenuated the mechanical allodynia in the rodent models of neuropathic pain [17, 27]. Consistently, in the present study, we further found that the STAT3 activation mediated the Nav1.6 upregulation in DRG neurons and mechanical allodynia following L5-VRT. Furthermore, p-STAT3 immunoreactivity was localized in DRG neurons expressing Nav1.6, which implies the likelihood that STAT3-mediated Nav1.6 upregulation occurred within the same DRG neurons. It is well-known that activated STAT3 can translocate to the nucleus to regulate the cytokine and chemokine expression [28, 29]. The present study further illustrated that the enhanced interaction between p-STAT3 and p300 in the *Scn8a* promoter increased the histone H4 hyperacetylation and facilitated Nav1.6 expression in DRG induced by L5-VRT.

As a crucial inflammatory factor, TNF-α has the capability to increase the neuronal excitability and mediate the behavioral hypersensitivity in neuropathic pain [10]. Here, we found that the increase of TNF-α signaling promoted Nav1.6 expression in DRG, which potentially increased the neuronal excitability and was involved in mechanical allodynia induced by L5-VRT. Furthermore, previous studies showed that inflammatory cytokines often activated various downstream signaling pathways, thus participating in the pathogenesis of neurological disorders. For example, the overexpression of cytokine CCL2 can lead to a marked increase of neuronal phosphorylated STAT3 in L5 DRGs [30]. Here, we found that the expression of TNF-α, STAT3, and Nav1.6 was primarily overlapped in DRG cells, and incubation of TNF-α increased the p-STAT3 expression in the cultured DRG cells. Importantly, intraperitoneal injection of the TNF-α inhibitor thalidomide prevented the STAT3 activation, H4 hyperacetylation, and Nav1.6 upregulation induced by L5-VRT. These results suggested that upregulation of the cytokine TNF-α mediated the STAT3 activation following L5-VRT, which was consistent with a previous report that IL-1β or TNF-α treatment could induce STAT3 phosphorylation in osteoblastic cells [15]. Taken together, these data suggested that activation of the TNF-α/STAT3 pathway induced the epigenetic upregulation of Nav1.6 expression in DRG and contributed to neuropathic pain induced by L5-VRT.

## Conclusions

Altogether, our study showed that TNF-α, via activating STAT3, mediated epigenetic upregulation of Nav1.6 in DRG and contributed to L5-VRT-induced mechanical allodynia, which provided a novel potential target for the treatment of nerve injury-induced neuropathic pain.

## Abbreviations

DRG: Dorsal root ganglion
L5-VRT: Lumbar 5 ventral root transection
mRNA: Messenger RNA
shRNA: Short hairpin RNA
STAT3: Signal transducer and transcription activator 3
TNF-α: Tumor necrosis factor-α
